# Extranodal NK/T-Cell Lymphoma, Nasal Type, Presenting as Refractory *Pseudomonas aeruginosa* Facial Cellulitis

**DOI:** 10.1177/2324709617716471

**Published:** 2017-07-06

**Authors:** Erika Reategui Schwarz, Katerina G. Oikonomou, Megan Reynolds, Juliette Kim, Rajeev L. Balmiki, Stephanie A. Sterling

**Affiliations:** 1 New York University, New York, NY, USA; 2 NYU Lutheran Medical Center, Brooklyn, NY, USA; 3 Saint George’s University, West Indies, Grenada

**Keywords:** lymphoma, extranodal NK-T-cell/pathology, Epstein-Barr virus infections/virology, United States, ENKL, humans

## Abstract

Extranodal natural killer T-cell lymphoma, nasal type (ENKL), formerly called lethal midline granuloma or angiocentric T-cell lymphoma, is a predominantly extranodal non-Hodgkin lymphoma characterized by vascular damage, necrosis, and an association with Epstein-Barr virus. In the United States, it is more frequently seen in Asian, Asian Pacific Islander, and Hispanic descent populations and is more prevalent in males in their fifth decade. Clinical presentation of NK nasal lymphoma most commonly involves epistaxis; obstruction; discharge; destructive mass in sinuses, palate, and nose; and skin ulceration. These symptoms can mimic invasive fungal infections and other sinonasal disorders. Furthermore, ENKL has a broad cytologic spectrum and induces a mixture of inflammatory cells, causing difficulty in establishing the diagnosis, especially in initial biopsies. We present a case of refractory *Pseudomonas aeruginosa* facial cellulitis in a young woman whose treatment course was complicated by septic shock and resistance to multiple antibiotics, resulting in a delayed diagnosis of ENKL nasal type.

## Introduction

Extranodal natural killer T-cell lymphoma, nasal type (ENKL), formerly called lethal midline granuloma or angiocentric T-cell lymphoma, is a predominantly extranodal non-Hodgkin lymphoma characterized by vascular damage, necrosis, and an association with Epstein-Barr virus (EBV).^1^ The term is designated as “NK/T” (instead of “NK”) due to some cases showing a cytotoxic T-cell phenotype (CD56-, TCR gene rearrangement).^2^

NK/T lymphoma is a rare entity more prevalent in Asia and Latin America (3% to 10%) as opposed to the United States and Europe (<1%).^3^ In the United States, it is more frequently seen in Asian, Asian Pacific Islander, and Hispanic descent populations and is more prevalent in males in their fifth decade.^4,5^

Clinical presentation of NK nasal lymphoma most commonly involves epistaxis; obstruction; discharge; destructive mass in sinuses, palate, and nose; and skin ulceration, and its presentation can mimic invasive fungal infections and other sinonasal disorders.^5^

Systemic symptoms like fever, hemophagocytosis, and disseminated intravascular coagulation may also be present. This disease is often localized to the upper aerodigestive tract at presentation, but may disseminate to skin, soft tissue, gastrointestinal tract, testis, and cervical lymph nodes. Occurrences outside the nasal cavity vary in presentation depending on the site of involvement; however, bone marrow involvement is uncommon and is associated with aggressive NK-cell leukemia.^6^

We present a case of refractory *Pseudomonas aeruginosa* facial cellulitis in a young woman whose treatment course was complicated by septic shock and multidrug antibiotic resistance, resulting in a delayed diagnosis of ENKL nasal type.

## Case Presentation

A 23-year-old Hispanic woman presented to the emergency department (ED) with persistent and worsening edema, erythema, tenderness, and warmth of the right nares and periorbital area associated with purulent nasal drainage. The patient had multiple ED visits over a 6-week period for her condition. She was initially diagnosed with severe sinus infection and was discharged on amoxicillin/clavulanate potassium, then switched to cephalexin 500 mg every 6 hours and clindamycin 300 mg every 8 hours for inadequate response. The patient was hospitalized on her third ED visit, and initiated on empiric intravenous (IV) vancomycin plus ceftriaxone. Computed tomography (CT) scan at the time showed right paranasal and inferior periorbital/premaxillar soft tissue swelling with subcutaneous sinus fat induration. On her fifth hospital day, culture of her nasal drainage grew *P aeruginosa*. She was discharged on oral ciprofloxacin 750 mg twice a day, based on antibiogram sensitivities.

**Figure 1.**
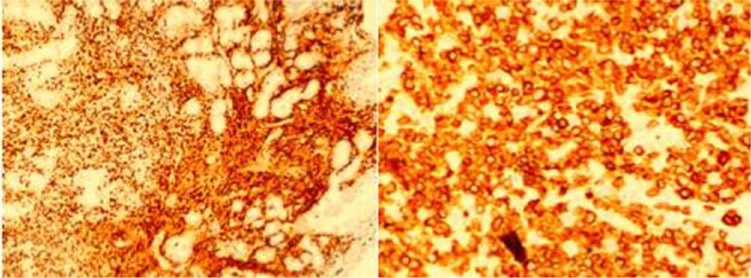
Right premaxillary mass immunohistochemistry: (a) Immunohistochemistry CD3+ MP ×10; (b) Immunohistochemistry CD3+ HP ×40.

The patient returned to the hospital 1 week after completion of her oral antibiotic therapy for worsening of skin erythema, right facial edema, and nasal discharge. Vital signs on admission were the following: temperature, 103°F; pulse, 133 beats per minute; blood pressure, 82/40; respiratory rate, 30 per minute. Laboratory tests were significant for white blood cell count, 4200/µL; lactic dehydrogenase, 387 IU/L; and lactic acid, 1.6 mmol/L. She was admitted to the intensive care unit with septic shock secondary to nonresolving facial cellulitis, received aggressive fluid resuscitation, vasopressors, and was started on IV vancomycin 1 g every 8 hours, gentamycin 210 mg daily, and meropenem 1 g every 8 hours. Magnetic resonance imaging of the face and orbits disclosed right nasal abscess and the patient underwent debridement in the operating room. The deep wound cultures grew multidrug-resistant *P aeruginosa* sensitive to meropenem; she was continued on this antibiotic. Antifungal coverage with amphotericin was initially added but then discontinued on tissue culture results. Pathology from nasal tissue was diagnostic for ENKL, nasal type. Immunohistochemical studies showed malignant cells CD3+, CD20−, and weakly cytoplasmic CD2+ and CD56+ (Figure 1).^6^ In situ hybridization for Epstein-Barr virus–encoded small nuclear RNAs (EBER) showed patchy positivity. Epstein-Barr virus antigen antibody (EBNA AB) was 4.64 U/mL, EBV viral capsid antigen (VCA) IgG was 4.93 U/mL, EBV VCA IgM of <0.91 U/mL. EBV quantitative viral load showed 3162 copies/mL.

The patient continued to spike fevers despite clinical improvement throughout her hospitalization. Subsequent nasal cultures showed *P aeruginosa* growth, increasingly resistant to antibiotics. Polymyxin 130 mg daily was added to meropenem 1 g every 8 hours on return of positive synergy testing. Staging CT scan of chest, abdomen, and pelvis and bone marrow microscopic examination were negative for metastatic disease. On clinical exam, the patient’s facial swelling continued to worsen, now with extension to the left face. Repeat nasal discharge cultures 10 days later revealed 2 different strains of *P aeruginosa* were growing, one extensively drug resistant and the other sensitive to her current antibiotic regimen. Antibiotic therapy was switched to ceftolozane/tazobactam 1.5 g every 8 hours. Chemoradiation was initiated 10 days later with a resultant deeffervescence and marked improvement in the diffuse bilateral facial swelling. Patient was discharged home from the hospital after completing a 4-week total IV antibiotic course. A posttreatment positron emission tomography (PET)/CT scan performed 3 months after revealed changes concerning for progression of systemic disease. She underwent a biopsy of a left thigh swelling, which was positive for ENKL, and commenced systemic chemotherapy at a nearby cancer center and completed 4 cycles of SMILE regimen (dexamethasone, methotrexate, ifosfamide, L-asparaginase, etoposide). Posttreatment PET/CT scan showed no evidence of disease and EBV quantitative polymerase chain reaction showed an undetectable copy number. Patient is currently undergoing evaluation for autologous stem cell transplant.

## Discussion

ENKL is a rare disease in the United States, though its prevalence is increasing, especially among Hispanic and Asian Pacific Islander populations.^5^ Men are affected more frequently, usually within the fifth decade of life, unlike our young female patient.

NK lymphoma has been reported to present as epistaxis; obstruction; discharge; destructive mass in sinuses, palate, and nose; and skin ulceration. Compared to other documented cases, septic shock is an uncommon presentation (Table 1).

**Table 1.** Review of NK Lymphoma Case Reports: Presentation, Treatment, and Outcome Comparison.

Author	PMID	Age	Location	Presentation	Treatment	Outcome
Termote et al^7^	24831171	45	● Left lower eyelid	● Painless swelling	● CHOP + radiation	● Died 5 months after diagnosis
		20	● Left upper and lower eyelid	● Painless swelling	● SMILE + DHAP + DEXA BEAM + radiation + autologous stem cell transplantation	● Died 9 months after diagnosis
		55	● Right upper and lower eyelid	● Painless swelling of the right eye and double vision	● SMILE + intrathechal methotrexate-cytarabine-hydrocortisone	● Died 35 months after diagnosis
Kim and An^8^	24621697	58	Right-sided orbit	Swelling and pain	Dexamethasone, ifosfamide, and etoposide regimen	Survived at the time of publication of case report
Marchino et al^3^	24317101	67	Left orbit	Exophthalmos, pain, swelling, and limited extrinsic ocular motility	CHOP + SMILE	Died 10 months after diagnosis
Pine et al^9^	23387455	52	Left orbit	Swelling, proptosis, vision loss, and diplopia	Methotrexate/cytarabine and L-aspariginase combination + radiation	Died 5 months after diagnosis
Kunami et al^10^	20622489	72	Left forearm	Painless swelling	SMILE + radiation + amputation	Survived without progression 2 years after amputation
Jia and Sun^11^	15359650	62	Right orbit	Swelling and purulent nasal drainage	Unavailable	Unavailable
Huang et al^12^	14719563	22	Left side of face	Swelling nasal obstruction	Cisplatin, cytosine arabinoside, and methotrexate + surgery	Died 7 months after diagnosis

Abbreviations: CHOP, cyclophosphamide, hydroxydaunorubicin, vincristine, and prednisone; SMILE, steroid = dexamethasone, methotrexate, ifosfamide, L-asparaginase, etoposide; DHAP, dexamethasone, high-dose cytarabine, cisplatin; DEXA BEAM, dexamethasone, carmustine, etoposide, cytarabine, and melphalan.

In our patient, it was unclear if septic shock was secondary to facial cellulitis or if the release of cytokines/interleukins from the tumor burden played a role on presentation. ENKL diagnosis was done from deep wound tissue biopsy from the right nares and paranasal sinuses biopsy. ENKL has a broad cytologic spectrum and induces a mixture of inflammatory cells, causing difficulty in establishing the diagnosis, especially in the initial biopsies. Miyake et al reported a case series in which up to 5 biopsies were required to achieve a definitive diagnosis.^13^ Out of 23 patients with ENKL treated at Ohio State University and 2 patients at the University of Colorado, a median time of symptoms to diagnosis of 5 months was established.^5^ Twelve of these patients required more than one diagnostic biopsy leading up to 36 months in delay of diagnosis.^5^

In our case, the initial biopsy did not show malignancy but the second biopsy showed malignant cells CD3+, CD20−, and weakly positive for cytoplasmic CD2 and CD56. In situ hybridization for EBER showed patchy positivity. Haverkos et al reported a consistent association of ENKTL-NT with EBV.^5^ In a 6-year prospective study, Au et al found that in all cases of EBV-positive lymphomas, EBV DNA paralleled the clinical course, with EBV DNA becoming undetectable at remission and remaining elevated in refractory disease.^14^ High levels of EBV DNA at presentation (>7.3 × 10^7^ copies/mL) were significantly associated with an inferior overall survival. In the NK cell lymphomas subgroup (the largest cohort), EBV DNA was correlated with disease stage and lactate dehydrogenase.^14^ For this reason, EBV viral load should be trended.

**Figure 2.**
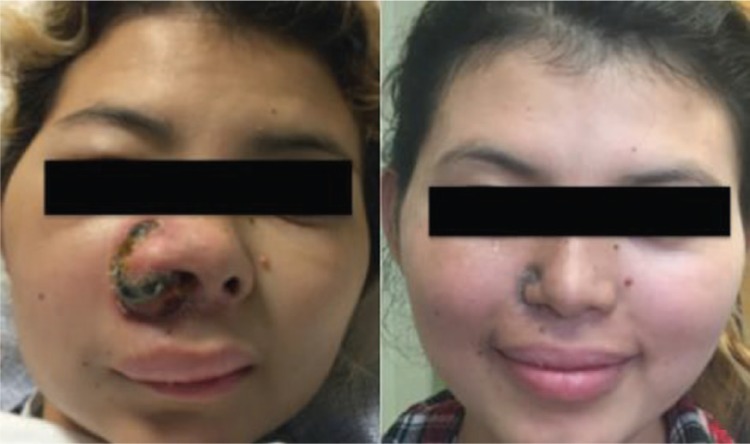
Right nares lesion during hospitalization course and after discharge: (a) Initiation of chemotherapy; (b) Three months after discharge.

Our patient’s EBV quantitative viral load was 3162 copies/mL. Her fevers resolved after radiotherapy and chemotherapy was initiated, although a new antibiotic regimen with ceftolozane/tazobactam was started few days prior. Therefore, it remains unclear if clinical improvement was attributed to the new antibiotic, to chemo/radiotherapy, or to the combination of the two. Although ceftolozane/tazobactam is not a conventional antibiotic used for cellulitis, in our case it was associated with initial limitation of local disease and improvement in cosmetic sequelae (Figure 2). Ceftolozane/tazobactam has been reported to successfully treat respiratory and intraabdominal infections secondary to multidrug-resistant *Pseudomonas* spp; however, to our knowledge, ours is the first reported case where this antibiotic cleared a cellulitis infection (cultures on patient discharge were negative).

Combined radiotherapy and chemotherapy is the standard treatment in early stage NK/T-cell lymphoma. Despite the initial response to radiotherapy alone, a high relapse rate of around 50% is reported due to possible systemic dissemination in nasal NK/T-cell lymphoma initially considered localized. Prognostic factors for NK/T-cell lymphoma are not yet fully established: biological parameters such as circulating EBV DNA and interim PET/CT findings should be considered in the construction of future prognostic models.^2,4^ In EBV-positive lymphomas, plasma EBV DNA is valuable as tumor biomarker and prognostication; however, ENKL has a poor outcome in general.^14^

## Conclusion

Early diagnosis of ENKL nasal type proves challenging due to the fact that its presentation mimics other sinonasal disorders and its broad cytological spectrum affects the accuracy of initial biopsies. Internists who encounter prolonged, complicated cases of facial cellulitis, sinusitis, or pharyngitis refractory to conventional treatment, especially in Asian, Asian Pacific Islander, or Hispanic populations and in the presence of EBV should consider testing for NKTCL, nasal type, in order to prevent rapid disease progression. Given the low level of suspicion for this neoplasm, diagnosis is often delayed because the biopsy specimen is necrotic or because bacterial and fungal stains are incorrectly interpreted as evidence that the primary process is an invasive bacterial or fungal sinusitis, rather than lymphoma, leading to repeated, but unsuccessful, courses of antimicrobial therapy.^5^

This delay in diagnosis may result in increased risk of extension of the disease compromising survival and cosmetic damage. Our case exhibits an unusual and life-threatening presentation of ENKL with superimposed bacterial infection leading to septic shock. Detecting the primary disease and starting chemoradiation along with ceftolozane/tazobactam ultimately yielded clinical improvement.
